# Metastasis inhibition by BRMS1 and miR-31 replacement therapy in claudin-low cell lines

**DOI:** 10.22038/IJBMS.2019.35674.8500

**Published:** 2020-02

**Authors:** Samila Farokhimanesh, Mehdi Forouzandeh Moghadam, Marzieh Ebrahimi

**Affiliations:** 1 Department of Medical Biotechnology, Faculty of Medical Sciences, Tarbiat Modares University, Tehran, Iran; 2 Department of Biotechnology, Science and Research Branch, Islamic Azad Unversity, Tehran, Iran; 3 Department of Stem Cells and Developmental Biology at Cell Science Research Center, Royan Institute for StemCell Biology and Technology, ACECR, Tehran, Iran

**Keywords:** Breast cancer, BRMS1, miR 31, Neoplasm metastasis, Replacement therapy

## Abstract

**Objective(s):**

The growing trend of research demonstrates that dynamic expression of two metastasis repressor classes (metastasis suppressor genes and anti-metastatic miRNA) has a close relationship with tumor invasion and metastasis. Using different strategies, it was revealed that cellular levels of miR-31 and Breast cancer Metastasis Suppressor1 (BRMS1) protein, which are among the most significant modulators of metastasis, have a correlation with the cell’s capability for invading and metastasizing; cells containing higher levels of miR-31 or BRMS1 were less metastatic. This project was carried out to determine whether the combinations of miR-31 and BRMS1 genes are able to enhance the capability of repressing the claudin-low breast cancer cell (MDA-MB-231) invasion.

**Materials and Methods:**

This study used a restoration-based approach by miR-31 mimic and optimized BRMS1 gene sequences, which were cloned into a chimeric construct and transfected to the MDA-M231cells.

**Results:**

Our data revealed that the simultaneous expression of anti-metastasis miR and metastasis suppressor might inhibit migration and invasion in MDA-MB-231 cells efficiently.

**Conclusion:**

This combinatorial use of anti-metastatic miR and gene suggests a new therapeutic intervention for metastasis inhibition in MDA-MB-231.

## Introduction

Among metastasis suppressors, researchers especially focused on BRMS1 because of the different respective anti-metastatic performances. This molecule basically functions through changing the SIN3: histone de-acetylase (HDAC) chromatin remodeling complexes. BRMS1 can influence various processes involved in metastasis (such as adhesion, migration, invasion, programmed cell death, angiogenesis, gap junctional connection, cytoskeleton remodeling, and increasing immune recognition) through changing this complex. This functional diversity is likely the logic related to robust antimetastatic effect of BRMS1 (1-3). BRMS1 also makes a bridge between metastasis inhibition and metastamiR modulation via up-regulating metastasis-inhibiting miRNAs (miR-146a, miR-146b, and miR-335) and down-regulating metastasis-enhancing miRNAs (miR-10b, miR-373, and miR-520c). BRMS1 is the only metastasis inhibitor that is able to regulate transcriptome, post-transcriptome, and proteome at molecular levels (4, 5).

Although it seems that most metastamiRs contribute essentially to invading and migrating cancer cells (6), just a few metastamiRs involved in numerous phases of the metastatic cascade are known currently. miR-31, is a pleiotropic miRNA (7) which enhances apoptosis (through protein kinase Cɛ) (8, 9) , affects cytoskeleton remodeling (via WAVE3, an actin cytoskeleton re-modeling protein, which has a high expression in the developed phases of breast cancer) (10), regulates integrins (multiple α subunit partners such as α2, α5,αV, and of β subunit, β1 and β3 integrins) (11), and down-regulates SATB2 (particular AT-rich sequence-binding protein 2 that was contributed to the gene transcription and chromatin re-modeling) (12). In addition, all miRNA target scan databases have introduced RDX, MMP16, GNA13, PRKCE, ITGA5, Fzd3, MPRIP, and RhoA as potential targets for miR-31 (13, 14).

Restoration of metastasis inhibitor levels (e.g., miRNAs or genes) in invasive cancer cells, in particular, as they function pleio-tropically, indicates an influential treatment choice for metastatic cancer. Since pleiotropic genes or miRNA target multiple genes/paths, re-expression of a single gene or miRNA to the non-affected tissues would provide a greater treatment impact in comparison with the medicines following the one-medicine-one-target pattern (15). This research revealed that restoration of expressing antimetastatic miR-31 and BRMS1 did not impact *in vitro* cellular viability or proliferation, while it highly decreased *in vitro *invasion or migration in MDA-MB-231 cells. Thus, combining both metastasis suppressors can further suppress metastasis process efficiently.

## Materials and Methods

***Generating DNA constructs***

First, retrieving the mature miR-31 sequence was performed via miRBase. Afterward, top and bottom oligo sequences of miR-31 were developed according to the kits’ instruction and chemically synthesized (Fazapajooh, Iran). Next it was annealed by μM200 concentration of each top and bottom strand, 10X oligo annealing buffer, RNase, and DNase-free water, and thereafter cloned within pcDNA 6.2-GW/EmGFPmiR following manufacturer’s protocols to produce pcDNA 6.2-GW/EmGFPmiR/miR-31 (pc.miR31). The oligonucleotide sequences for miR-31 were:

*Top strand*

5′TGCTGTTTCTAGGGATGCTGATGCTGGTTTTGGCCACTGACTGACCAGCATC

AATCCCTAGAAA-3′

*Bottom strand*

5’CCTGAGGCAAGATGCGCATAGCTGTCAGTCAGTGGCCAAAACAGCTATGCCAGCATCTTGCCTC 3’

In order to recognize possible target genes of miR-31, TargetScan, miRanda, miRDB, and PicTar databases were used. In order to reach maximum levels of expressions, GenScript (Genscript Corporation Piscataway; NJ, USA) was used to optimize the BRMS1 gene sequence. Next, the optimized gene was cloned into pcDNA 6.2-GW/EmGFPmiRneg (pc.neg) control plasmid (Invitrogen, Carlsbad, CA, USA) (pc.BRMS1) and pcDNA 6.2-GW/EmGFPmiR-31 (pc.miR-31.BRMS1) through SalI and DraI restriction enzymes (Roche Applied Science; Castle Hill; NSW, Australia). The resulting chimeric vector causes (co-cistronic) co-expression of BRMS1in the position of EmGFP and miRNA-31 included in a murine miR-155 setting under human cytomegalovirus promoter. Sequencing was used to confirm the accuracy of each chimeric vector. 

***Cell culture***

*Cell lines*

Cell lines were obtained from ATCC, including MCF-7 (low invasiveness breast cancer cells, luminal breast cell line), MDA-MB-231 (high intrusiveness triple-negative breast cancer cells, claudin-low cell line), and MCF-10A (normal breast cell line). 

*Tissue collection and Single-cell suspensions procurement*

Reduction mammoplasty materials were inserted into sterile falcons with Dulbecco’s modified Eagle’s medium/Ham’s F-12 (DMEM/F12) with 5% fetal bovine serum (FBS), 100 U per ml penicillin, 100/~g per ml streptomycin at 4 °C; then, it was transported to the laboratory. The tissue was processed to separate epithelial from stromal constituents. Skin and fatty regions were dissected, and the residual tissue was slowly lacerated with opposing scalpels. The tissue was transported to a sterile 50-ml conical centrifuge falcon with a magnetic stir bar, and enzyme digestion mixture involving 2% FBS, 2X antibiotic, 200 U/ml crude collagenase (Sigma, Missouri, USA), and 100 U/ml hyaluronidase (Sigma, Missouri, USA). Enzymatic digestion was performed at 37 ^°^C by gently agitating via a hybridizer. After overnight incubation, suspension was pelleted at 80 x g for 30 sec. Fatty layers were eliminated, pellet (pellet A) was maintained, and supernatants were transported to a novel falcon and centrifuged at 200×g for 3 min and repeated for pellet B. Both pellets were re-suspended in pre-warmed enzyme digestion mix (Trypsin/EDTA) in 15-ml clear plastic centrifuge tubes. Then trypsin was neutralized by Hank’s buffer plus 2% FBS; centrifuging of tubes was done at 350×g for 5 min. The supernatants were eliminated, and 2 ml pre-warmed Dispase (Sigma, Missouri, USA) with a concentration of 5 mg/ml and DNaseI with a concentration of 5 mg/ml were added to pellets, and pipetting was performed for 1 min. the suspensions then were diluted by Hank’s buffer and filtered through 40 µm nylon mesh. Centrifugation was done at 350×g for 5 min, and supernatant was discarded. The culture medium (DMEM/F12) was added to the pellet.

*Flow cytometric analysis of surface expression level of CD44* ^+^ */CD24* ^-^ *in claudin-low MDA-MB-231 cells*

Trypsinization of MCF-7, MDA-MB-231, and normal breast cells (separated from Reduction mammoplasty material) was performed and suspended in PBS at 1×10^5^ cells/ml density. Propidium iodide at the ultimate concentration of 2 μg/ml was poured into the cells to gate viable cells. The cells were stained with fluorophore-conjugated antibodies versus 2 human cell surface markers, that is, fluorescein isothiocyanate (FITC) conjugated CD24 (StemCell Technologies Inc, Vancouver, Canada) and phycoerythrin (PE) conjugated CD44 (eBioscience; San Diego; CA: USA), and their isotype controls at the concentrations suggested by manufacturers. Then, incubation was done at 4 ^°^C in the dark for 30 to 40 min. When incubation was done, the cells were washed 3 times to eliminate unbound antibody. 530/30 nm (FL1) and 585/42 (FL2) bandpass filters and logarithmic amplification were used to collect fluorescence emission. Data were analyzed using the Flowjo software package. 

*Transfections*

5^ 4 ^cells of MDA-MB-231 and MCF-7 cell lines were placed in 24-well plates and incubated overnight. Both cell lines were transiently transfected by pc.neg, pc.miR-31, pc.BRMS1, and pc.miR-31.BRMS1, using (lipofectamine 2000 Invitrogen, Carlsbad, CA) for subsequent experiments. 48 hr post-transfection, green fluorescent protein (GFP), and flowcytometry were used to determine efficacy.

*MTT assay*

The cell density of MDA-MB-231 cells was set to 3×10^2^ cell/ml for single-cell suspension. Seeding a volume of 500 ml single-cell suspension was established within 24-well plates. When the cells achieved 80% confluency, they were transfected with regard to the earlier description. Each transfection was done three times. 24 hr post-transfection, the cells were trypsinized and re-suspended in 1 ml medium, and then these cells were diluted five times. Seeding was performed in 96-well plates. When the cells were grown for 24, 48, and 72 hr at 37 ^°^C in 5% CO_2_ incubator, their incubation was performed with 10 µl methyl thiazolyl tetrazolium (MTT, 0.5 mg/ml, Sigma, St Louis, Missouri, USA) per well. Following cell incubation with MTT for four hours, an inverted microscope was employed for observing formazan crystallization. The culture medium was aspirated. Then, 100 ml dimethyl sulfoxide (Sigma, St Louis, Missouri, USA) was used to dissolve crystals per well. An ELISA (enzyme-linked immunosorbent assay) plate reader at 570 nm wavelength (reference wavelength 630 nm) was used to measure the optical density.

*Extracting RNA and real-time PCR*

RNeasy mini kit (Qiagen, Hilden, Germany) was employed to extract total RNA from three cell lines before and 48 hr after transfection. The Revert Aid First Strand cDNA Synthesis Kit (Fermentas; Thermo Fisher Scientific, Waltham, MA, USA) was applied for random selection of cDNA from total RNA. Real-time PCR assays were done three times through Sybr Premix Ex Taq II (Takara; Tokyo, Japan) on a Rotorgene 3000 series PCR machine (Corbett Research, San Francisco, USA) using following primers for endogenous BRMS1, OCT-4, and Survivin, respectively:

BRMS1F.5’-AGC TCT GAA TGG TGG GAT GAC-3’

BRMS1R.5’-CAC GAT GTA TGG GCC AGA AAC-3’

Oct-4 F.5’-GTTCTATTTGGGAAGGTATTC-3′

Oct-4 R. 5′-ACTGGTTCGCTTTCTCTTTC-3′

Survivin F. 5′-CCACCGCTACTCTACATTC-3′

Survivin R. 5′-CTTTCTCCGCAGTTTCCTC-3′

Rotorgene software and PCR machine were used to collect and analyze data. Moreover, the comparative quantification characteristic of the Rotorgene software was selected for determining the relative levels of expressions. Each mRNA quantification datum was normalized to βactin, and changes in the folds of expression were determined by the comparative Ct (ΔΔCt) technique.

*Extracting miRNA and real-time PCR*

Total RNA, with efficient recovery of small RNAs, was drawn out from 3 cell lines before and 48 hr after transfection using the miRCURY RNA isolation kit (Exiqon, Denmark), and miRCURY LNA™ Universal RT miRNA PCR, polyadenylation, and cDNA synthesis kit (Exiqon, Denmark) were used for reverse transcription of 2 μl of total RNA in 10 μl reactions. 4 ul of cDNA diluted 50x and 1 ul of forward and reverse primer were assayed into 10 ul PCR reactions for detection of the mature form of miR-31, through miRCURY LNA Universal RT microRNA PCR Kit and LNA microRNA Primer Sets, based on the instructions of miRCURY LNA™ Universal RT miRNA PCR. DNA and RNA spike-ins were considered in the qPCR and RT phases. Negative control with no reverse transcriptase enzyme has also been treated and included in the profile as the specimens. Rotorgene software was applied to determine Ct values and generate amplification and melting curves. Each assay was checked in terms of different melting curves. T_m_ was evaluated to check whether it is within the defined ranges for assaying. Only assays determined with 5 Ct’s lower than the negative control and with Ct < 37 were considered to analyze data. Those data, which did not meet the criteria, were excluded from any additional analyses. Raw Ct values were corrected by the average amplification efficiency.

U6 small nuclear RNA was applied as an internal control for normalizing RNA input in a real-time RT-PCR assay, and the Pfaffl method was used for specifying relative quantities of expressions. 

*Scratch assay*

MCF-7 and MDA-MB-231 cells were plated in 6-well plates. The cells were cultured in a medium with 10% FBS to close confluence of the cell monolayer. Afterward, a linear wound was generated in the mono-layer via scratching through a plastic pipette tip. PBS was used to wash the mono-layer two times for removing debris or separated cells. The wounded mono-layer was incubated for 24 hr. The cell migrated to the wound site was detected via an inverted microscope at times 0 and 24 after scratching.

*Transwell migration assay (TMS)*

TMS was done on the basis of the company guidelines through transwell cell culture chamber units (Millipore, Billerica, MA, USA) with 8-μm pore size polycarbonate membranes. The migration rates were determined by counting the migrated cells to the bottom of the filters in various fields under a microscope at 200×. Assays were iterated three times.

*Tranwell invasion assay*

The cell invasion assay was conducted under similar conditions with migration assay, with the exception of coating transwell cell culture chambers with matrigel. The migrated cells were counted by an inverted microscope. A similar process was fundamentally proceeded for measuring transwell cell migration towards a chemo-attractant via replacement of Matrigel-coated transwell chambers with the un-coated transwell chambers. 

***Statistical analyses***

Each experiment was conducted no less than 3 times for all groups. Outputs were written as the mean SD. The student’s t-test was employed for determining statistical significance. The asterisk implies it is significant (*P*<0.05). 

## Results

***Claudin-low MDA-MB-231 cells have a high proportion of CD44*** ^+^ ***/CD24*** ^-^ ***cells***

**Figure 1:**
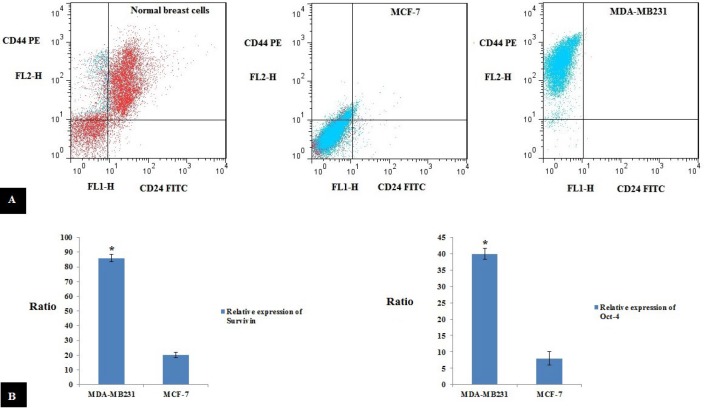
A) Normal breast cells (separated from reduction mammoplasty material), MCF-7 and MDA-MB-231 flow cytometry. The percent of CD44^+^/CD24^-^ cell population is 11% in normal breast cells, 25% in MCF-7 cells, and 88% in MDA-MB-231 cells. B) Relative expression of Oct-4 and Survivin in MDA-MB-231 and MCF-7. (*P-value<0.05)

For testing the hypothesis that MDA-MB-231 cell lines possess a high proportion (>90%) of CD44^+^/CD24^-^ cells, MDA-MB-231, MCF-7 cell lines, and normal breast cells (separated from reduction mammoplasty material) were assessed via flowcytometry to investigate the expression of CD24 and CD44 surface proteins, which have a main relation with breast cancer stem cell markers. CD44^+^/CD24^-^ cells percentage in MDA-MB-231, MCF-7, and normal breast cells were 95%, 8%, and 6-7%, respectively (Figure 1A).

***The expression levels of Oct-4 and Survivin are significantly high in claudin-low MDA-MB-231 cells in comparison with luminal MCF-7 cells***

For further characterization of MDA-MB-231 cells, expressing putative stem cell marker, Oct-4, and anti-apoptotic protein, Survivin were analyzed. Outputs demonstrated that the levels of Oct-4 and Survivin expressions in MDA-MB-231 cells are considerably higher (5 fold and 4.3 fold, respectively) than MCF-7 cells (Figure 1B).

***Anti-metastatic miR-31 and BRMS1 expressions were lower in claudin-low MDA-MB-231 cells in comparison with luminal MCF-7cells***

**Figure 2:**
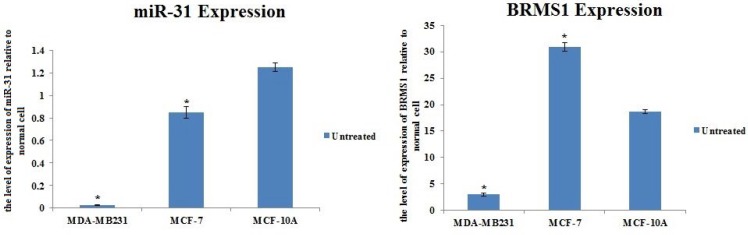
A) The level of miR-31 expression in MDA-MB-231, MCF-7, and MCF-10A. B) The level of BRMS1 expression in MDA-MB-231, MCF-7, and MCF-10A. (*P-value < 0.05)

For proving that miR-31 levels decreased in MDA-MB-231 cells, this study made a comparison of miR-31 levels in MDA-MB-231, MCF-7, and MCF-10A. The comparison revealed that MDA-MB-231 cells have significantly lower levels of miR-31 than MCF-7 and MCF-10A. The expression of miR-31 in MCF-10A was 52 fold more than MDA-MB-231 and almost 1.47 fold more than MCF-7 cells (Figure 2A). Moreover, BRMS1 level was assessed prior to any treatment. Evaluation outputs indicated that expression level of BRMS1 was 10 fold lower than MCF-7 and almost 6 fold lower than MCF-10A (Figure 2B).

***miR-31 and BRMS1 up-regulation in claudin-low MDA-MB-231 cells did not affect these cells proliferation***

**Figure 3:**
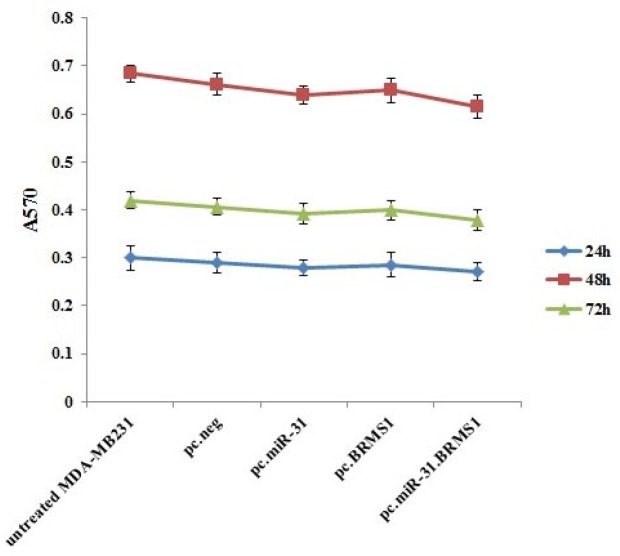
Proliferation rates of untreated and pc.neg, pc.miR-31, pc.BRMS1, and pc.miR-31.BRMS1 transfected MDA-MB-231 after 24, 48, and 72 hr

The growth rate of MDA-MB-231 cells was contrasted between untreated pc.neg, pc.miR-31, pc.BRMS1, and pc.miR-31.BRMS1 transfected cells. At 24, 48, and 72 hr post-transfection, the rate of cell proliferation was almost identical in 4 groups. Up-regulation of BRMS1 and miR-31 did not have any effects on the proliferation of MDA-MB-231cells (Figure 3).

***Restoring miR-31 and BRMS1 expressions prevents the migration of claudin-low MDA-MB-231 cells***

**Figure 4:**
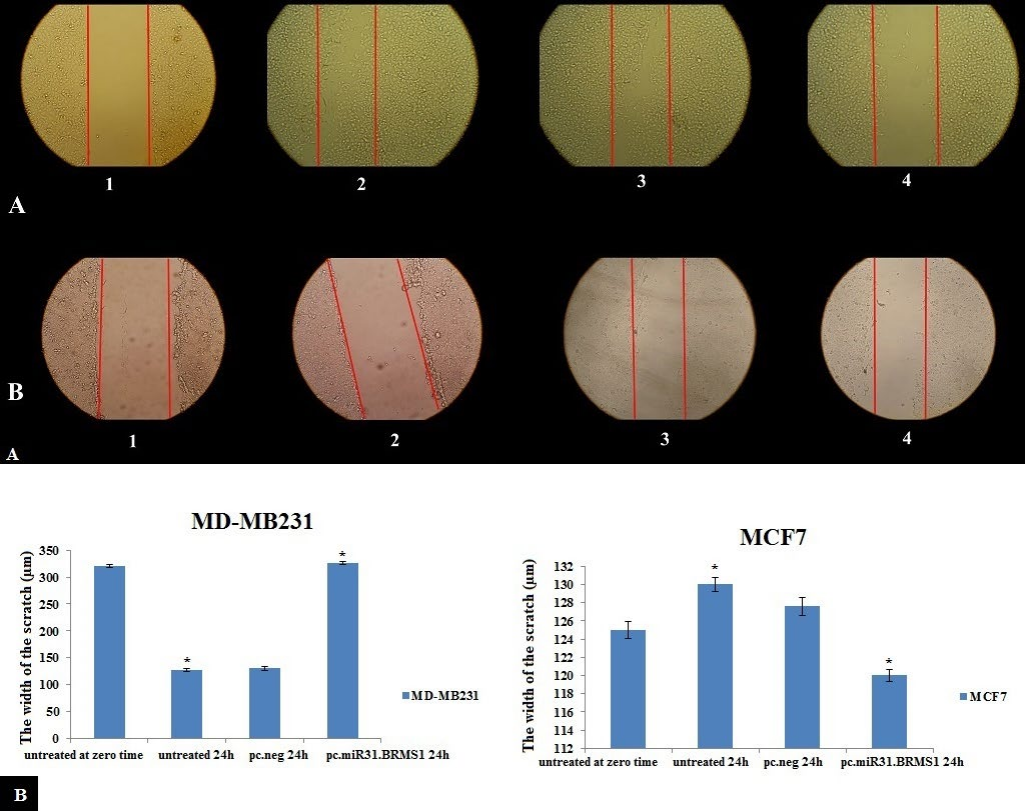
(A) Scratch assay in MDA-MB-231 at zero time [(a1). Scratch assay in untreated MDA-MB-231 after 24 hr (a2). Scratch assay in pc.neg transfected MDA-MB-231 after 24 hr (a3). Scratch assay in pc.miR-31.BRMS1 transfected MDA-MB-231 after 24 hr (a4). Scratch assay in MCF-7 at zero time (b1). Scratch assay in untreated MCF-7 after 24 hr (b2). Scratch assay in pc.neg transfected MCF-7 after 24 hr (b3). Scratch assay in pc.miR-31.BRMS1 transfected MCF-7 after 24 hr (b4)]. (B) The width of the scratch at different times in MDA-MB-231. (C) The width of the scratch at different times in MCF-7. (*P-value < 0.05)

A wound-healing assay was done for assessing the impacts of miR-31 and BRMS1 on cell migration. According to Figures 4A, B, and C), scratching test showed that 48 hr after transfecting, the scratch width in pc.miR-31.BRMS1 transfected MDA-MB-231 group was almost the same as untreated MDA-MB-231 at zero time (326.3 µm). This width was significantly wider than the pc.neg transfected MDA-MB-231 group (127.1 µm) and the untreated group (130 µm). As expected, the untreated MCF-7 at zero time, the untreated MCF-7 after 24 hr, pc.neg group, and the pc.miR-31.BRMS1 transfected MCF-7 had no significant difference in scratch width, and the range of changes was between 121-130 µm. 

***miR-31 and BRMS1 suppress migrating and invading claudin-low MDA-MB-231 cells***

**Figure 5:**
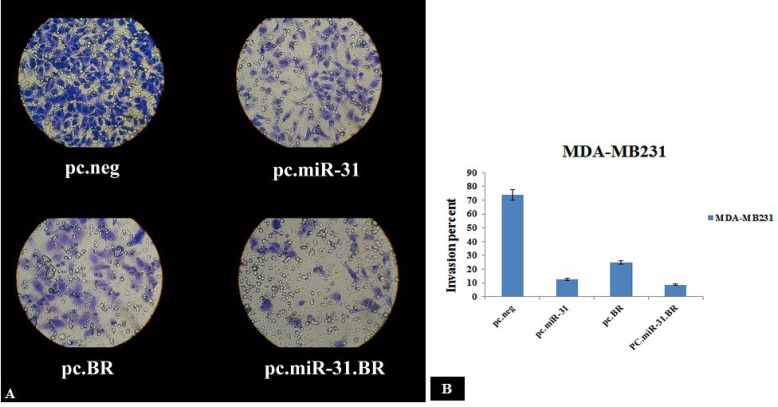
A) Invasion assay in pc.neg, pc.miR-31, pc.BRMS1, and pc.miR-31.BRMS1 transfected MDA-MB-231 after 24 hr. B) Invasion percent in p c.neg, pc.miR-31, pc.BRMS1, and pc.miR-31.BRMS1 transfected MDA-MB-231 after 24 hr. (*P-value < 0.05)

For determining whether miR-31 and BRMS1 regulate MDA-MB-231 cell invasion and metastasis or not, this study carried out *in vitro* gain-of-function analyses via ectopic expression of miR-31 and BRMS1 in MDA-MB-231 and MCF-7 cells. Transwell migration and invasion assays were performed on pc.neg, pc.miR-31, pc.BRMS1, and pc.miR-31.BRMS1 cells. We observed that ectopic expression of miR-31 and BRMS1 considerably (not less than 8.5 fold reduction) inhibited invading MDA-MB-231 cells in Transwell assays with Matrigel, and declined the cell migration in Transwell assays with no Matrigel (Figures 5A, B).

## Discussion

Replacement treatments have emerged as a highly hopeful treatment strategy for cancer especially for its most deadly aspect, metastasis (16). Such therapy includes reintroducing a molecule (e.g., gene or miRNA molecules) for restoration of a loss-of-function, and in this way, it provides a novel ground and chance for exploring remedial potentials of metastasis inhibitors (16, 17). Since replacement treatment gives back gene products already found in normal tissues, it minimizes the toxicity. In addition, most molecules with differential expression are inhibited in metastatic tumor tissues in comparison with healthy tissues. This fact proposes that the possibility of being a tumor or metastasis suppressor is more than being oncogene (18). In this regard, replacement of pleiotropic molecules has gained much attention because their mechanisms of action are in line with our recent opinion of metastasis as a pathway disease. Considering these points, pleiotropically acting BRMS1 and miR-31 were selected for replacement therapy. As many replacement therapies are more sufficiently effective with a combinatorial approach (19), we have devised a combinatorial therapeutic intervention by using two potent metastasis suppressors including metastasis suppressor gene and metastasis suppressor miRNA, which act pleiotropically to inhibit metastasis. Both of the inhibitors function on the selective phases of metastatic cascade. BRMS1 inhibits metastasis by repressing several phases in the cascade via regulating different metastasis-related genes and metastasis-regulatory microRNAs (20).

To evaluate the effectiveness of this combinatorial strategy, the MDA-MB-231 cell line, which was enriched with stem cell-like features and has a high invasive potential, was selected. Our results were in concordance with reports regarding the high proportion (>90%) of CD44^+^/CD24^- ^cells in MDA-MB-231 cell lines (21-23). For further characterization of MDA-MB-231 cells, expressing Oct-4 (putative stem cell marker) and anti-apoptotic protein Survivin (24) were analyzed. Results indicated that MDA-MB-231 cells had higher expression rates of Oct-4 and Survivin in comparison to non-metastatic cells. Endogenous expressions of miR-31 and BRMS1 molecules were assessed with the intention of confirming their down-regulated expression. It was hypothesized that such molecules sustain the differentiated mode of the organs. Expression patterns of these molecules correspond to a similar procedure during developing, differentiating, and cancer. Expression levels of the molecules will be low during development, rise to the highest level after differentiation to the adult state, and ultimately decrease in cancer. Previous research performed on miR-31 and BRMS1 independently found that restoration of the molecule expression returned the normal phenotypic characteristic. In support of our results, previous reports have demonstrated that an inverse correlation exists between BRMS1 and miR-31 expression, disease development, and lengthy survival of people suffering from breast cancer (25-27). Our anti-metastatic construct restored the expression of these molecules. Up-regulating miR-31 and BRMS1 suppresses cell invasion and migration in MDA-MB-231 cells. This study found that ectopic expression of BRMS1 and miR-31 molecules mainly influenced the invasive procedure instead of the rapid growth of MDA-MB-231 cells.

## Conclusion

We obtained credible proof that re-expressing miRNA-31 and BRMS1 suppresses cell migration and invasion in MDA-MB-231 cells by modulating different molecules engaged in metastatic cascade. Hence, the notion of the use of the chimeric replacement treatment constructs might be applied as a potential treatment for breast cancer metastasis. 
